# Acceptor dependent catalytic properties of GH57 4-α-glucanotransferase from *Pyrococcus* sp. ST04

**DOI:** 10.3389/fmicb.2022.1016675

**Published:** 2022-10-06

**Authors:** Jong-Hyun Jung, Seungpyo Hong, Eun Jung Jeon, Min-Kyu Kim, Dong-Ho Seo, Eui-Jeon Woo, James F. Holden, Cheon-Seok Park

**Affiliations:** ^1^Radiation Research Division, Korea Atomic Energy Research Institute, Jeongeup, South Korea; ^2^Department of Molecular Biology, Jeonbuk National University, Jeonju, South Korea; ^3^Department of Chemical and Biomolecular Engineering, Korea Advanced Institute of Science and Technology (KAIST), Daejeon, South Korea; ^4^Department of Food Science and Technology, Jeonbuk National University, Jeonju, South Korea; ^5^Korea Research Institute of Bioscience and Biotechnology (KRIBB), Daejeon, South Korea; ^6^Department of Microbiology, University of Messachusetts, Amherst, MA, United States; ^7^Department of Food Science and Biotechnology and Institute of Life Science and Resources, Kyung Hee University, Yongin, South Korea

**Keywords:** acceptor binding site, disproportionation, 4-α-glucanotransferase, hyperthermophilic archaea, glycoside hydrolase family 57, *Pyrococcus*

## Abstract

The 4-α-glucanotransferase (4-α-GTase or amylomaltase) is an essential enzyme in maltodextrin metabolism. Generally, most bacterial 4-α-GTase is classified into glycoside hydrolase (GH) family 77. However, hyperthermophiles have unique 4-α-GTases belonging to GH family 57. These enzymes are the main amylolytic protein in hyperthermophiles, but their mode of action in maltooligosaccharide utilization is poorly understood. In the present study, we investigated the catalytic properties of 4-α-GTase from the hyperthermophile *Pyrococcus* sp. ST04 (PSGT) in the presence of maltooligosaccharides of various lengths. Unlike 4-α-GTases in GH family 77, GH family 57 PSGT produced maltotriose in the early stage of reaction and preferred maltose and maltotriose over glucose as the acceptor. The kinetic analysis showed that maltotriose had the lowest KM value, which increased amylose degradation activity by 18.3-fold. Structural models of PSGT based on molecular dynamic simulation revealed two aromatic amino acids interacting with the substrate at the +2 and +3 binding sites, and the mutational study demonstrated they play a critical role in maltotriose binding. These results clarify the mode of action in carbohydrate utilization and explain acceptor binding mechanism of GH57 family 4-α-GTases in hyperthermophilic archaea.

## Introduction

Starch is one of the most abundant natural polymers, which is a major carbon and energy source of living organisms. To utilize the starch, diverse starch-degrading and starch-modifying enzymes occur in microorganisms, plants, and animals. The enzyme 4-α-glucanotransferase (4-α-GTase, EC 2.4.1.25) has a vital role in α-glucan (starch, maltodextrin, and glycogen) metabolism (Takaha et al., 1993; Lee et al., 2006). The enzyme produces maltooligosaccharide of various lengths by mediating disproportionation reaction that transfers a 1,4-α-glucan (donor) to a new C-4 position of the other glucan molecule (acceptor) or displaces a 1,4-α-glucan from a glucan molecule.

4-α-GTases are classified into the three GH families, 13, 57, and 77, based on their amino acid sequence similarities (Godany et al., 2008; Kuchtova and Janecek, 2015). They are not homologous but share the same catalytic activities that facilitate disproportionation, cyclization, hydrolysis, and coupling of glucans. GH77 is the most common 4-α-GTases family of plants, bacteria, and archaea (especially, in Crenarcheota; Kuchtova and Janecek, 2015). GH13 and GH57 families are observed in hyperthermophiles. A GH13 family 4-α-GTase was found in *Thermotoga maritima* (Roujeinikova et al., 2002), and six GH57 family 4-α-GTases were identified in *Thermococcus litoralis*, *Thermococcus kodakarensis* KOD1, *Pyrococcus furiosus*, *Dictyoglomus thermophilum, Thermococcus onnurineus, and Archaeoglobus fulgidus* (Laderman et al., 1993; Jeon et al., 1997; Tachibana et al., 2000; Nakajima et al., 2004; Labes and Schonheit, 2007; Kaila and Guptasarma, 2019).

In *P. furiosus* and *T. litoralis*, the 4-α-GTase is main amylolytic enzyme that enables the micro-organisms to utilize maltose and starch, and they are upregulated in response to these substrates (Imamura et al., 2003; Lee et al., 2006). It is well established that 4-α-GTases have strong transglycosylation activity and produce maltooligosaccharides of various lengths. However, GH57 family enzymes differ from GH77 and GH13 in their core structure and substrate interaction. GH57 has a (β/α)_7_ barrel core while GH77 and GH13 have a (β/α)_8_ barrel core (Imamura et al., 2003). GH57 has been established as smaller α-amylase family containing various enzyme specificity, such as α-amylase, branching enzyme, α-galactosidase, glucan maltohydroase, and 4-α-GTase (Janecek and Martinovicova, 2020). Until now, 4,250 GH57 sequences were registered in Carbohydrate Active enZyme database (CAZy; http://www.cazy.org/; Drula et al., 2022). The bioinformatics studies showed that there are five conserved regions (CSRs) reflecting enzyme specificities in GH57 family (Martinovicova and Janecek, 2018). Although their specificities are similar with other family enzymes, the GH57 family enzyme possesses its own enzymatic action mode. Especially, glucan maltohydrolase recognized and hydrolyzed maltose unit linked by α-1,4- and α-1,6-glycosidic bonding (Jung et al., 2014). And also, the amylase inhibitor, acarbose, binds to +3 to +1 glucose moiety binding sites of GH57 4-α-GTase, whereas it binds to the −1 to −3 sites of GH77 4-α-GTase (Przylas et al., 2000). Especially, GH57 4-α-GTase from *T. onnurineus* exhibited not only transferase activity, but also exo-amylase activity toward maltose(Kaila and Guptasarma, 2019). It suggested that the maltooligosaccharide metabolism *via* GH57 would be different from those of GH77 and GH13.

In *Thermococcale*, such as *Pyrococcus* and *Thermococcus*, glucan phophorylase, α-glucosidase, and two GH57 enzymes, 4-α-GTase and glucan maltohydrolase, were the common maltodextrin-hydrolyzing enzymes. Glucan maltohydrolase and α-glucosidase produced glucose from maltose and maltotriose, respectively. Glucan phosphorylase generated glucose-1-phosphate from maltooligosaccharides longer than maltotetraose (Mizanur et al., 2008). Glucan maltohydrolase is an unique enzyme to the order *Thermococcales*, and it hydrolyzes both the α-1,4 and α-1,6 linkages between glucose units (Jeon et al., 2014; Jung et al., 2014). Its action is limited by maltose concentration and feedback inhibition, which suggested that *Thermococcales* utilize the oligosaccharides with different manners. To our knowledge, the roles of GH57 family 4-α-GTase enzymes and its mechanisms of action are poorly understood despite of its important role in short-maltooligosaccharides utility of the genus.

In the present study, we investigated the enzymatic properties of a GH57 4-α-GTase of *Pyrococcus* sp. ST04 and demonstrated that has an unique maltooligosaccharide catalysis mechanism differs from that of GH77 4-α-GTases, marked by difference in acceptor binding.

## Materials and methods

### Bacterial strains and reagent

*Pyrococcus* sp. ST04 is a heterotroph hyperthermophilic archaeon that optimally grows at 92°C. Its growth conditions were described by Oslowski et al. (2011) and in the supplementary method (Supplementary Tables S1, S2). *Escherichia coli* DH10B [F^−^ *ara*D139 (*ara leu*)7697 *lac*X74 *gal*U *gal*K *rps*L *deo*R Φ80*lac*ZΔM15 *end*A1 *nup*G *rec*A1 *mcr*A (*mrr hsdRMS mcrBC*)] served as the host for DNA cloning. *E. coli* BL21-CodonPlus(DE3)-RP strain [F^−^ *ompT hsdS*(r_B_^−^ m_B_^−^) *dcm*^+^ Tet^r^ *gal* λ(DE3) *end*A Hte *argU proL* Cam^r^] was the host target protein expression. The *E. coli* strains were grown in Luria-Bertani (LB) medium (BD Bioscience, Franklin Lakes, NJ, United States) supplemented with ampicillin (100 μg/ml) and chloramphenicol (34 μg/ml).

PrimeSTAR DNA polymerase and DNA-modifying enzymes containing restriction endonuclease and ligase were purchased from TaKaRa Bio Inc. (Kusatsu, Shiga, Japan) and New England Biolabs (Beverly, MA, United States), respectively. Substrates containing maltooligosaccharides ranging in size from maltotriose to maltoheptaose were obtained from Wako Pure Chemical Industries Ltd. (Osaka, Japan). Glucose, maltose, and amylose were purchased from Sigma-Aldrich Corp. (St. Louis, MO, United States). The pGEM T-easy vector (Promega, Madison, WI, United States) and the pET-21a(+) vector (Novagen, Darmstadt, Germany) were used for PCR cloning and recombinant protein expression.

### Growth curve and growth rate in various carbon sources

To compare the growth rate in the different carbon source, growth mediums were made in 100 ml-serum bottles described in Supplementary materials. Briefly, 0.5% starch and maltose were used as carbon sources; and tryptone was not added in growth medium as *Pyrococcus* sp. ST04 can use it as a carbon source. Cell concentrations were calculated through cell counting using a Petroff-Hausser counting chamber and a microscope. The specific growth rate (k) was calculated by linear regression analysis from the exponential portions of the growth curves. All experiments were performed in triplicate.

### Determination of amylolytic activity of *Pyrococcus* sp. ST04 crude extract

To examine the amylolytic activity of cell extract, 500 ml culture of *Pyrococcus* sp. ST04 grew at 92°C for 18 h was harvested and filtered through filter paper (Whatman) to remove particle such as sulfur. The filtrate was centrifuged at 12,000 × *g* for 20 min. The pellets were resuspended and washed in 50 mM sodium phosphate pH 7.0 buffer and disrupted by sonication (4 × 3 min, output control 4, 28% duty cycle). The amylolytic activity of *Pyrococcus* sp. ST04 crude extract toward α-linked substrates was determined by thin layer chromatography (TLC) analysis. The prepared crude extract was mixed with soluble starch (0.5%), β-cyclodextrin (0.5%), malotriose (10 mM), and maltopentaose (10 mM) dissolved in 50 mM sodium phosphate buffer (pH 7.0) and reacted at 80°C for 12 h, respectively. The hydrolysis products were analyzed using TLC method described in the “Thin layer chromatography.”

### Construction of the pET-PSGT expression vector

To amplify *Py04_0423* gene (GenBank accession no. AFK22025.1) from the genome, the oligonucleotides ST_AMT_F, 5′- ATTAATATGGTGAACTTCATATTTGGGATTCACAA-3′ and ST_AMT_R, 5′- CTCGAG AACCTCTAAAAACTTTATCCTTATTTTTTCTTC-3′ were used as primers. PrimeSTAR DNA polymerase was used to obtain low-error PCR products, and the PCR was conducted under the following conditions: initial denaturation step, 40 s at 94°C; 20 cycles of 40 s at 94°C, 40 s at 57°C, 3 min at 72°C for amplification; and 5 min elongation at 72°C. The PCR products were purified with an Axygen Gel extraction kit (Axygen Scientific Inc., Union City, CA, United States) for cloning. The purified PCR products were ligated with pGEM-T-easy vector. The sequences of the fragment ligated with the vector were analyzed and confirmed. The amplified 1.9-kb fragment had extra terminal restriction enzyme sequences. For gene cloning, a fragment ligated with a T-vector was digested with the restriction endonucleases *Ase*I and *Xho*I. The fragment was inserted into the expression vector pET-21a(+) vector digested with *Nde*I and *Xho*I. The constructed expression vector pET-PSGT had a six-His tag in the frame at the *C*-terminal.

### Recombinant enzyme expression and purification

The constructed expression vectors were transformed into *E. coli* CodonPlus (DE3)-RP strain for efficient heterologous protein expression. The strain harboring pET-PSGT was cultured in 250 ml LB broth supplemented with ampicillin (100 μg/ml) and chloramphenicol (34 μg/ml) at 37°C until OD_600_ = 0.55. Recombinant protein expression was induced by adding 1 mM isopropyl β-d-1-thiogalactopyranoside (IPTG) and incubating at 37°C for 20 h.

Incubated cells were collected by centrifugation at 4,000 × *g* for 20 min and suspended in 20 ml lysis buffer [50 mM NaH_2_PO_4_, 300 mM NaCl, and 10 mM imidazole (pH 7.5)]. The pelleted cells were disrupted by sonication (4 × 5 min, output control 4; 40% duty cycle) with a VC-600 (Sonics & Materials Inc., Newtown, CT, United States), and centrifuged at 12,000 × *g* for 20 min. To remove the thermolabile proteins in the *E. coli* host strain, heat treatment was conducted at 70°C for 20 min. After centrifugation, the crude extracts containing the recombinant proteins with *C*-terminal six-His tags were applied to nickel-nitrilotriacetic acid (Ni-NTA) resins (Qiagen, Hilden, Germany). The resins were rinsed with 20 ml washing buffer [50 mM NaH_2_PO_4_, 300 mM NaCl, and 20 mM imidazole (pH 7.5)] and eluted with elution buffer [50 mM NaH_2_PO_4_, 300 mM NaCl, and 250 mM imidazole (pH 7.5)].

### Multiple sequence alignment analysis

For multiple sequence alignment analysis, 10 sequences of GH57 4-α-GTase were obtained from NCBI database^1^ and CAZY database.^2^ Sequence alignment was performed using Clustal Omega (Sievers and Higgins, 2021), and visualization was implemented using GeneDoc 2.7.

### Enzyme activity determination

#### Amylose degradation activity of recombinant PSGT

The amylose degradation activity of the recombinant PSGT (rPSGT) was determined by the iodine method. A reaction mixture (50 μl) containing rPSGT (0.097 mg/ml) and 0.5% (w/v) amylose dissolved in 90% (v/v) dimethyl sulfoxide (DMSO) in 50 mM sodium phosphate buffer (pH 7.0) was incubated at 85°C for 5 min. The reaction was stopped with 50 μl of 1 M HCl, and 1 M NaOH was added to neutralize the acid. A 100-μl aliquot was mixed with 900 μl iodine solution (0.2 g I_2_ and 2.0 g KI in 1 l H_2_O) at room temperature. Absorbance was measured at 660 nm, and 1 U amylose degradation activity was defined as the amount of enzyme hydrolyzing 1 μg amylose/min.

To measure amylose degradation activity in the presence of maltooligosaccharide acceptors, various acceptor concentrations were incubated with 0.5% (w/v) amylose dissolved in 90% (v/v) DMSO in 50 mM sodium phosphate buffer (pH 7.0) at 85°C for 5 min in the presence of 0.097 mg/ml rPSGT. The transglycosylation factor was defined as the ratio of the amylose degradation activity in the presence of the acceptor to the amylose degradation activity in the absence of the acceptor. All assays were conducted in triplicate, and the standard errors were calculated.

#### Kinetic analysis of rPSGT acceptor specificity

A kinetic analysis of the rPSGT acceptor specificity was determined by the iodine method. One hundred-microliter reaction mixtures containing various concentrations of each acceptor (glucose, maltose, and maltotriose in the range of 0.32–10 mM) and 0.5% (w/v) amylose as the substrate were incubated at 85°C for 2 min in the presence of 0.097 mg/ml rPSGT. The kinetic parameters of the reaction were calculated by a Lineweaver-Burk plot.

#### Hydrolysis pattern of rPSGT with various substrates

Substrate specificity of rPSGT toward various maltooligosaccharides from maltose to maltoheptaose was determined by TLC analysis. 10 mM of each substrate was incubated with 0.097 mg/ml of rPSGT at 80°C for 12 h. For time course analysis of disproportionation reaction, hydrolysates for 0–120 min were collected and visualized using TLC.

To determine hydrolysates distribution at initial reaction time, 0.097 mg/ml rPSGT was mixed with 1 mM of various maltooligosaccharides and incubated at 85°C for 2 min. The reaction was stopped with 150 mM NaOH. The hydrolysis products were identified and quantified using high-performance anion exchange chromatography (HPAEC) analysis.

The action pattern of rPSGT was examined using *p*NPG6. The 0.097 mg/ml rPSGT was mixed with 1 mM of *p*NPG6 and incubated at 85°C for 0–10 min. The products were identified using TLC analysis. The *p*NPG2–*p*NPG10 marker was made from pNPG6 using disproportionation reaction of TBGT (Jung et al., 2011, 2014).

### Site-directed mutagenesis

To find the amino acids interacting with the acceptor, hydrophobic aromatic residues located in the +2 and +3 subsites of the structure model with acarbose molecule were investigated. Based on the distance, three amino acid residues (Y181, F185, and W219) were finally selected as interacting factor candidates. To examine the function of these residues, Y181A, F185A, and W219A amino acid substitution mutants were constructed with a QuikChange site-directed mutagenesis kit (Agilent Technologies, Santa Clara, CA, United States). The mutations were introduced with mutagenic primers (Supplementary Table S3). Mutagenesis PCR was performed over 17 cycles of 95°C for 30 s, 55°C for 30 s, and 68°C for 8 min. The amplified products were digested with *Dpn*I and transformed into *E. coli* DH10B. Target sequence substitution was verified by sequencing.

### Structure model and docking simulation

The structure of PSGT was modeled by using SWISS-MODEL (https://swissmodel.expasy.org; Waterhouse et al., 2018) from its sequence and the structure of *T. litorallis* 4-α-glucanotransferase (PDB ID = 1K1Y, chain A), of which sequence identity to PSGT is 65%. The alanine mutation structures were generated by removing the atom coordinates for the mutation residues except the main chain and β-carbon atoms. The three dimension conformations of glucose (CID = 5,793), maltose (CID = 6,255), maltotriose (CID = 192,826), and maltotetraose (CID = 446,495) were retrieved from NCBI PubChem (Kim et al., 2019).

The receptor and ligand structures for the docking simulation were prepared by using AutoDock Tools (*prepare_ligand4.py* and *prepare_receptor4.py* scripts; Morris et al., 2009). Docking simulation was executed with AutoDock Vina (version 1.1.2; Trott and Olson, 2010) in the cube of length 30 Å centered at the active site (to the coordinate of N4A atom of acarbose in PDB ID = 1K1Y), with the following settings: *exhaustiveness* = 100 and *num_modes* = 20.

The positional coordinate of a ligand is determined by projecting the center of heavy atom coordinates of the ligand to the axis along the substrate binding site. The axis is defined as the vector between two acarbose atoms that lie along the substrate binding site (C4A and C1 atoms of the acarbose), and the origin of the coordinate is defined at the coordinate where the atom at the active site (N4A atom of the carbose) is projected onto the vector.

The sub-angstrom atomic coordinate match to the acarbose is evaluated as the percentage of ligand atoms matched to the acarbose atoms. The pairwise distances between the ligand and acarbose heavy atoms are calculated. The pairs with shortest distance are detected, and the atom is considered to be matched if the distance is shorter than 1 Å. The two atoms are removed from further procedure. The shortest distance detection is repeated until the match to carbose is evaluated for all ligand atoms.

### Analysis of the reaction products

#### Thin layer chromatography

Thin layer chromatography was performed on a Whatman K5F silica gel plate (Whatman, Kent, United Kingdom) after activation at 110°C for 10 min. Prepared samples were loaded onto a silica gel plate and placed in a TLC chamber containing 3:1:1 isopropanol:ethyl acetate:water for maltooligosaccharide analysis. The silica gel plate was oven-dried at 110°C and developed by rapid soaking in 0.3% (w/v) 1-napthiol and 5% (v/v) H_2_SO_4_ in methanol. The silica gel plate was dried and heated to 110°C in an oven for 10 min to visualize the reaction spots.

#### High-performance anion exchange chromatography

The reaction mixture was passed through a nylon membrane filter (0.2 μm pore diameter, Whatman, Kent, United Kingdom). HPAEC was performed using a CarboPac™ PA-1 (Dionex Co., Sunnyvale, CA, United States) and an electrochemical detector (ED40; Dionex Co.). The samples were eluted with a linear gradient from 100% buffer A (150 mM NaOH) to 35% buffer B (150 mM NaOH and 600 mM Na-acetate) at 1 ml/min over 35 min.

## Results

### Utilization of α-glucan by *Pyrococcus* sp. ST04

Members of the order *Thermococcales* can degrade cellulosic and amylolytic materials at high temperatures. The *P. furiosus*, well-known starch utilizing *Thermococcales*, harbors various GH13 family amylolytic enzymes. Strain ST04 is a heterotroph hyperthermophilic archaeon isolated from a deep-sea hydrothermal sulfide chimney on the Endeavour Segment of the Juan de Fuca Ridge in the northeastern Pacific Ocean (Oslowski et al., 2011). The genome sequence of strain ST04 (GenBank accession no. CP003534) showed that only three GH57 family amylolytic enzymes were observed, whereas GH13 family enzymes were not found (Supplementary Table S4; Jung et al., 2012). It is interesting that strain ST04 is known to produce H_2_S and H_2_ through utilizing carbon source, maltose (Oslowski et al., 2011).

**Figure 1:**
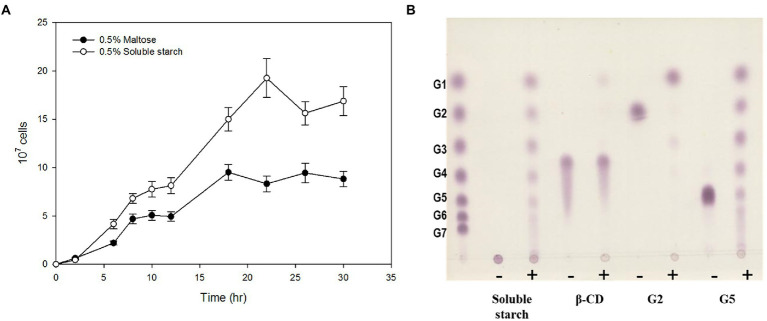
α-Glucan utilization by *Pyrococcus* sp. ST04. **(A)** Growth curves of *Pyrococcus* sp. ST04 on maltose (closed circles) and soluble starch (open circles). **(B)** Hydrolytic activity of ST04 crude extract on various substrates. Reactions were performed at 80°C for 12 h. (−) and (+) indicate presence and absence of crude extracts of *Pyrococcus* sp. ST04, respectively.

To investigate the ability of strain ST04 to grow on α-glucan, we separately measured its growth rate in the presence of maltose and starch. ST04 grew faster on starch (0.493 ± 0.021 h^−1^) than on maltose (0.332 ± 0.015 h^−1^). Moreover, its cell density on starch media was twice as much of that on maltose (Figure 1A). To determine the amylolytic activity of strain ST04, we incubated a crude extract of it with α-linked substrates, such as starch, maltose, cyclodextrin, and maltopentaose. The extract showed hydrolytic activity for α-glucans such as maltose and starch (Figure 1B). However, it could not break the cyclodextrin ring. Strain ST04 also exhibited strong disproportionation activity for maltopentaose and starch but not for maltose. Together, *Pyrococcus* sp. ST04 has strong utilization activity for α-glucans, which can be facilitated by 4-α-glucanotransferase (4-α-GTase).

### *Pyrococcus* sp. ST04 GH57 glucanotransferase identification and expression

**Figure 2:**
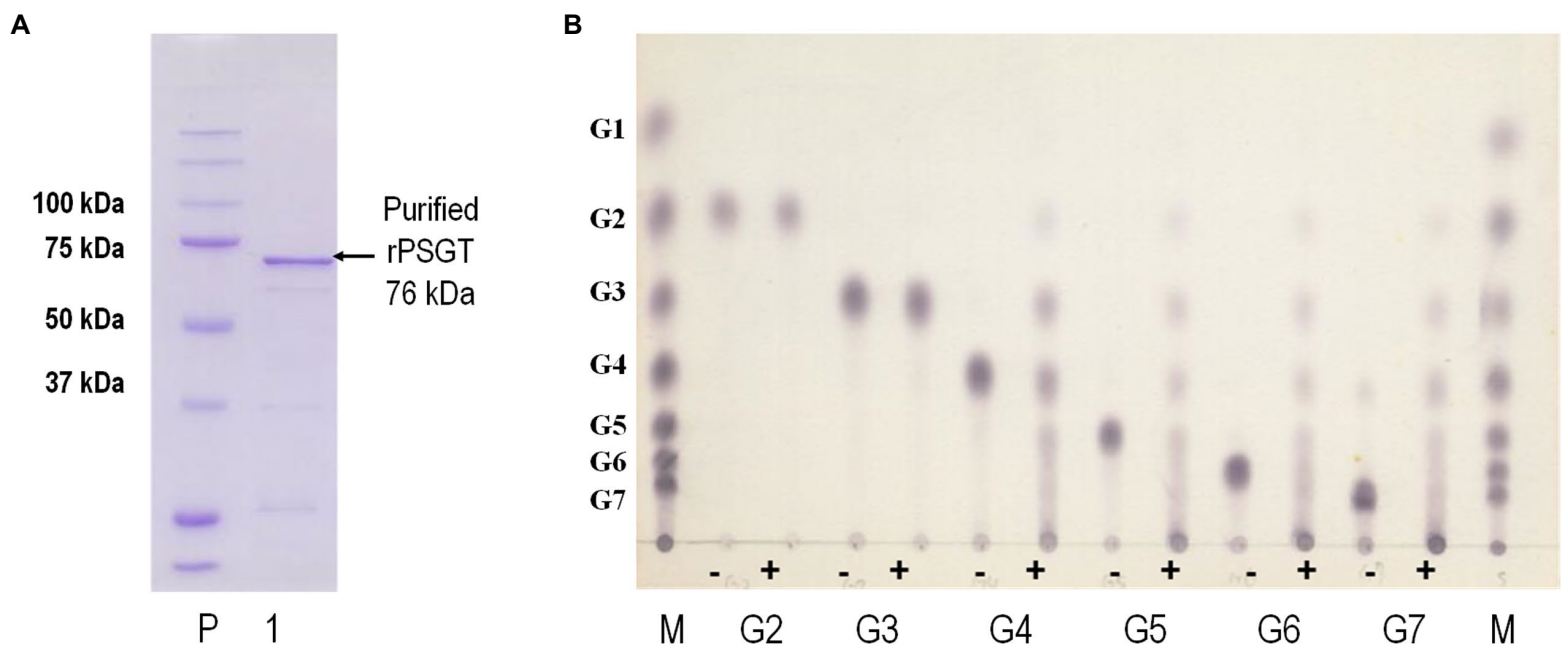
Biochemical characterization of recombinant PSGT (rPSGT). **(A)** SDS-PAGE of purified rPSGT; Lane P, protein ladder; Lane 1, purified band of rPSGT. **(B)** Substrate specificity of rPSGT toward various maltooligosaccharides from maltose to maltoheptaose. Each substrate (10 mM) was reacted with rPSGT at 80°C for 12 h. (−) and (+) indicate presence and absence of enzyme, respectively.

In the genome sequence of strain ST04, the gene encoding 4-α-GTase (Py04_0423, PSGT) was located between nucleotide position 427,657 and 429,588 and comprised 1,932 nucleotides encoding 644 amino acid residues. PSGT showed 75 and 65% sequence identity with the glucanotransferases from *P. furiosus* and *T. kodakarensis* KOD1, respectively (Tachibana et al., 2000; Tang et al., 2006). To investigate the enzymatic properties of PSGT, we cloned the gene, and recombinant PSGT (rPSGT) was expressed and purified using the pET-21a(+) vector and *E. coli* CodonPlue(DE3)-RP strain. Within a single SDS-PAGE band, a 70-kDa recombinant protein was observed, which corresponds to the calculated molecular mass of PSGT (76,002 Da; Figure 2A). Though rPSGT was expressed in *E. coli*, it was not denatured by heat treatment at 70°C. The effect of temperature on rPSGT activity was tested in the range of 40–99°C. Maximum activity was observed at 85°C and pH 7 when amylose was used as its substrate (Supplementary Figure S1).

### Maltooligosaccharide utilization by GH57 4-α-GTase

4-α-GTase catalyzes disproportionation, coupling, cyclization, and hydrolysis reactions. The enzyme is known to facilitate disproportionation of maltodextrin (Xavier et al., 1999; Lee et al., 2006). To investigate disproportionation activity, we incubated 10 mM of maltooligosaccharides, composed of 2–6 glucoses, with 0.097 mg/ml rPSGT at 80°C. rPSGT had disproportionation activity on maltotetraose (G4), maltopentaose (G5), maltohexaose (G6), and maltoheptaose (G7; Figure 2B). However, maltose (G2) and maltotriose (G3) were not favorable substrates of rPSGT. rPSGT seldom released glucose or maltose during disproportionation reactions. The predominant reaction products were maltotriose and longer maltooligosaccharides. This discovery was consistent with the slower growth rate of strain ST04 on maltose compared to the growth on starch. The inability of producing glucose is a unique property of PSGT, as other GH77 family 4-α-GTases mainly generate glucose and exhibit high hydrolysis activity on maltotriose (Kaper et al., 2007; Jung et al., 2011).

**Figure 3:**
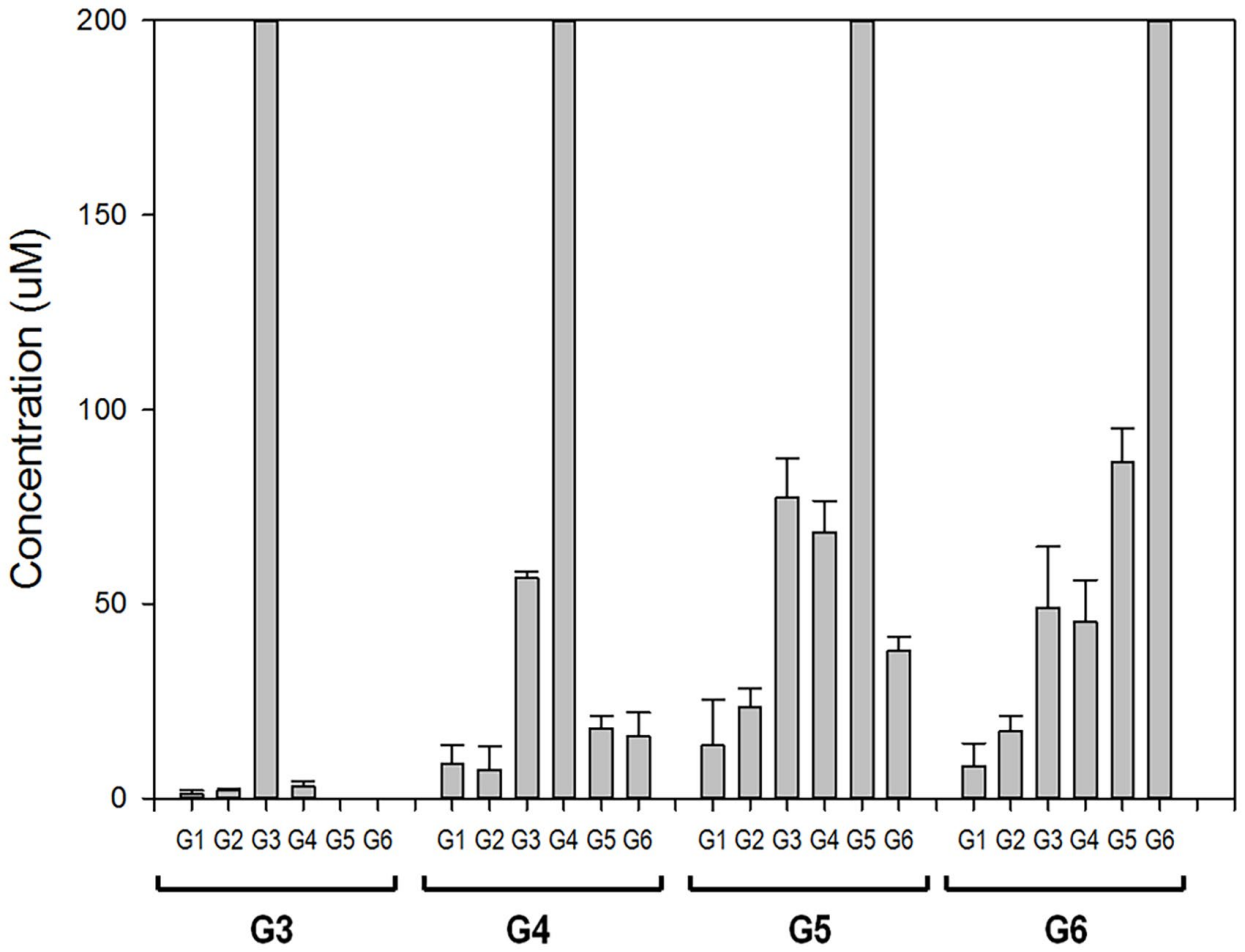
PSGT hydrolysis product distribution at initial reaction time. Reaction mixtures with 0.097 mg/ml rPSGT and 1 mM of each substrate were incubated at 85°C for 2 min.

To elucidate the reaction mechanism, we used high-performance anion exchange chromatography (HPAEC) to determine the profiles of maltooligosaccharides produced at the time of the initial enzymatic reaction. rPSGT had high activity on maltooligosaccharides longer than maltotriose and produced relatively low glucose and maltose yield (Figure 3). Maltotriose was the most abundant product at the initial enzymatic reaction of maltotetraose and maltopentaose, and it was the second abundant product of maltohexaose reaction. The products that are shorter than substrates by one glucose were also highly abundant. In contrast, little degradation reaction took place when maltose and maltotriose were used as substrates. Maltotriose was a major product of the reaction with maltotetraose, but little glucose was produced. These results suggest that rPSGT would hydrolyze a glucose from maltooligosaccharides that are longer than maltotriose and the glucose would be trapped in intermediate form, which directly used as a substrate of disproportionation.

### GH57 4-α-GTase mode of action

**Figure 4:**
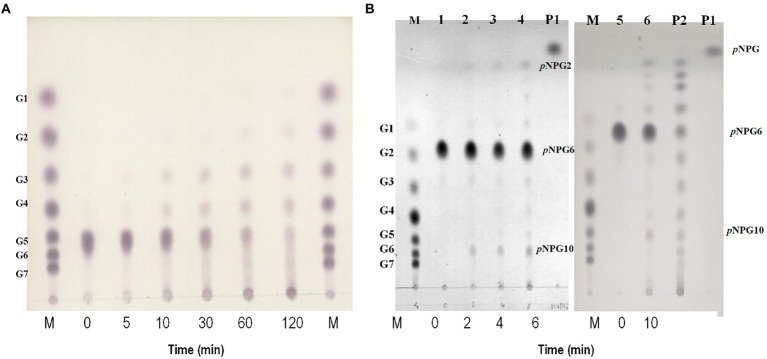
Hydrolytic action of rPSGT. **(A)** Time course analysis of disproportionation reaction pattern of rPSGT in the presence of maltopentaose. Reaction mixtures were incubated with 10 mM maltopentaose at 85°C for 0–120 min. **(B)** Hydrolytic action of rPSGT on 10 mM *p*NPG6. Reaction with 0.097 mg/ml rPSGT at 85°C for various time intervals (2–10 min). Lane M, G1–G7 standard; Lane P1, *p*NPG standard; and Lane P2, *p*NPG2-10 standard.

Maltotriose was produced by hydrolysis of all substrates longer than maltotriose, but maltotriose itself did not function as a substrate. To clarify the mode of action of GH57 4-α-GTase, we incubated maltopentaose with it and used TLC to determine its reaction products at various time intervals (Figure 4A). Maltotetraose and maltotriose were the major disproportionation reaction products. In contrast, neither glucose nor maltose was generated from the initial reaction. After 2 h, however, small quantities of glucose and maltose formed.

Disproportionation is a complex reaction and its mechanism is difficult to understand as the products of disproportionation can also be used as substrates of the reaction. To identify the binding site preference for maltooligosaccharides, the reaction can be monitored by using *p*-nitrophenyl-α-hexopyranoside (*p*NPG6) as the sole substrate. At the start of the reaction, *p*-nitrophenyl-maltopyranoside (*p*NPG2) and *p*-nitrophenyl-decapyranoside (*p*NPG10) were detected. Hence, while *p*NPG2 was released from the enzyme, maltotetraose remained at the substrate binding site and participated in *p*NPG10 synthesis. Over time, *p*NPG3 and *p*NPG4 were also produced, and their transglycosylation products *p*NPG9 and *p*NPG8, respectively, were generated (Figure 4B).

### Effects of the acceptor binding site on the GH57 4-α-GTase enzymatic reaction

Based on the foregoing results, we concluded that PSGT preferentially released oligosaccharides longer than maltotriose at the initial reaction time. It also suggested that PSGT may have an acceptor binding site, which has sufficient space to bind with oligosaccharides longer than maltotriose. We further investigated how glucose, maltose, and maltotriose binding affect the catalytic activity of the enzyme. For determination of acceptor specificity, the amylose degrading activity of the enzyme was measured in the presence of 5 mM of glucose, maltose, and maltotriose. The enzymatic activity levels were 6.8 ± 0.3, 18.2 ± 1.6, and 27.4 ± 2.3 mg amylose/min·mg, respectively. Amylose degradation was about 12.2 and 13.3-fold faster in the presence of maltose and maltotriose, respectively, than it was in the absence of acceptor molecules (Table 1). Acceptor specificity for maltotetraose was too difficult to measure since maltotetraose was also working as a substrate. It rapidly cleaved into glucose and maltotriose in the reaction.

**Table 1:** Amylose degradation activity and transglycosylation factors of rPSGT in the presence of acceptors.

	Specific activity (mg amylose/min·mg)	Transglycosylation factor^*^
No acceptor (amylose)	1.5 ± 0.2	–
Glucose	6.8 ± 0.3	4.6 ± 0.2
Maltose	18.2 ± 1.6	12.2 ± 1.4
Maltotriose	27.4 ± 2.3	18.3 ± 1.5

* Transglycosylation factor was calculated as the ratio of amylose degradation in the presence of acceptor (5 mM) to that in its absence.

**Figure 5:**
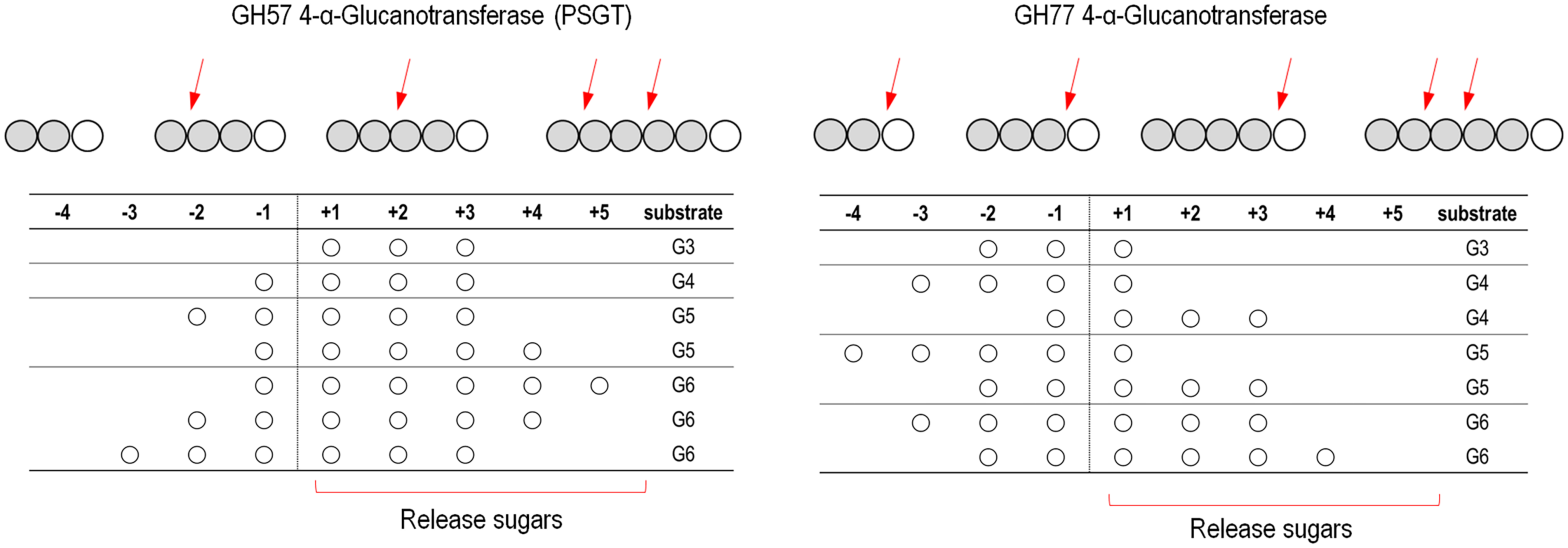
Schematic representation of the binding modes of maltooligosaccharides of GH57 (PSGT) and GH77 4-α-GTase. The numbering indicated that substrate binding subsites. The α-1,4-glucosidic bonding of substrates is cleaved between subsite −1 and + 1. Red arrow means dominant cleavage site of various substrates. The binding modes of GH77 4-α-GTase was represented based on the experimental data reported by Jung et al. (2011) and Kaper et al. (2007)

Kinetic analyses of acceptor molecule-dependent amylose degradation activity are summarized in Table 2. Maltotriose had the lowest *K*_M_ value (0.86 ± 0.06 mM) for the amylose degradation reaction. Thus, the PSGT acceptor binding site would be compatible with maltotriose. *K*_M_ for maltose (1.81 ± 0.19 mM) was lower than that for glucose (6.76 ± 0.68 mM). The efficiency ratio (*k*_cat_/*K*_M_) indicated that PSGT had the highest activity with maltotriose (Table 2). The results showed that sugar molecules shorter than maltotriose have a preferential affinity for acceptor binding sites over intermediate sites. In case of molecules longer than maltotriose, most molecules will bind on the subsite containing the acceptor binding site from +1 to +3 (Figure 5). In the reaction of GH57 PSGT with maltopentaose, most maltopentaose will bind on the subsite from −2 to +3 or −3 to +2 with high probability, which was a different pattern with other GH77 4-α-GTase that maltopentaose mainly binds on the subsite from −4 to +1 (Figure 5). We suggested a mechanism that would explain this activation in the Discussion section.

**Table 2:** Kinetic parameters of rPSGT for acceptor specificity.

Substrate	*K*_M_ (mM)	*k*_cat_ (min^−1^)	*k*_cat_ */K*_M_
Glucose	6.76 ± 0.68	1,141 ± 93	168 ± 14
Maltose	1.81 ± 0.19	2,008 ± 102	1,109 ± 56
Maltotriose	0.86 ± 0.06	2,635 ± 113	3,064 ± 131

### Roles of Y181 and F185 in acceptor binding

**Figure 6:**
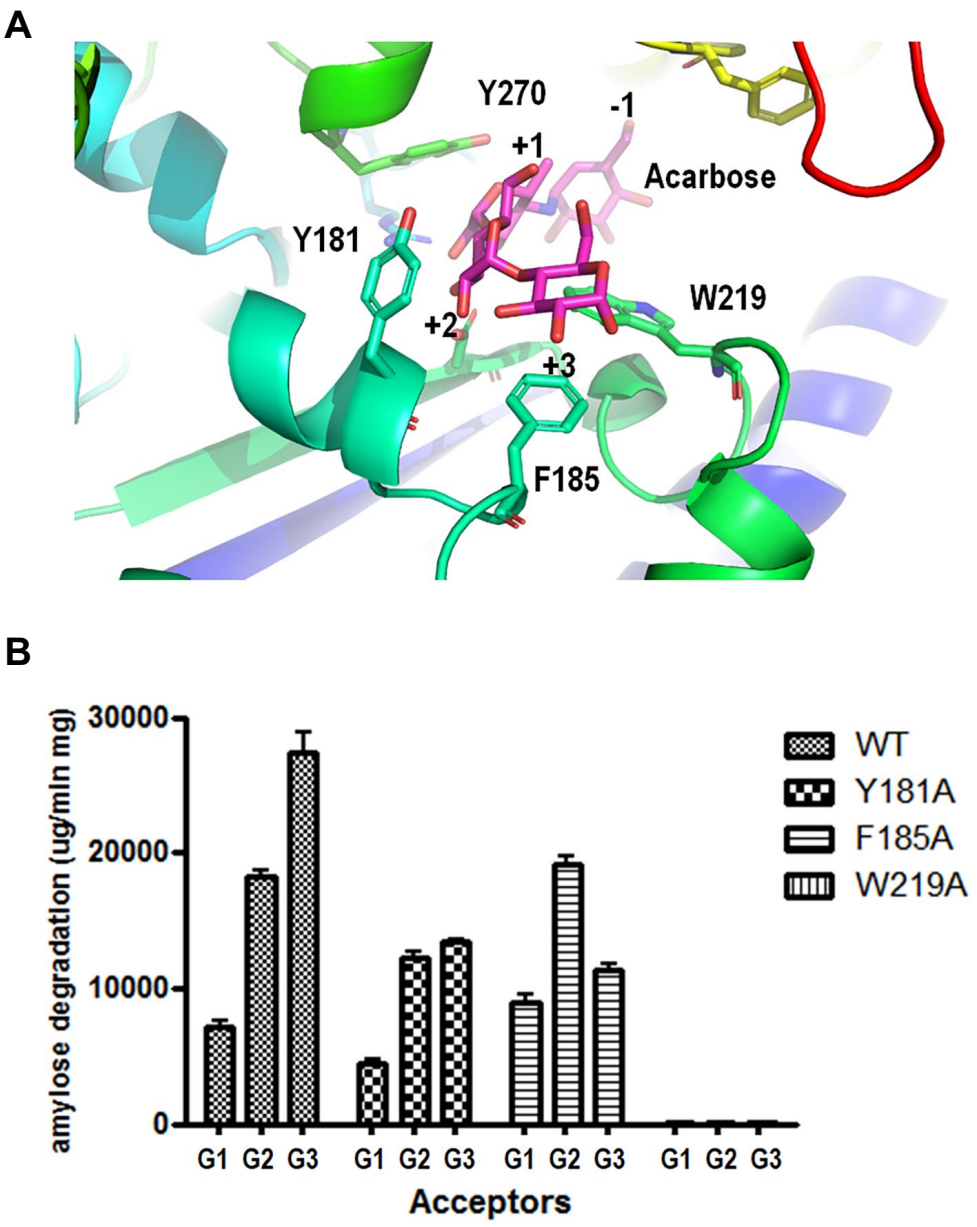
Effects of aromatic residues in the acceptor binding site of PSGT. **(A)** Structural model of PSGT superimposed with acarbose. **(B)** Amylose degradation activity of WT and mutant rPSGT in the presence of 5 mM glucose, maltose, or maltotriose as acceptor.

To identify the key amino acid residues of acceptor binding, we constructed a 3D homology model of PSGT based on the available crystal structure of 4-α-glucanotransferase from *T. litoralis* (PDB ID: 1K1Y). Modeling was performed using the SWISS-MODEL (65% identity). To identify the acceptor binding key amino acid residues of 4-α-GTases structures, acarbose was used as pseudo-substrate molecule (Przylas et al., 2000; Imamura et al., 2003; Kaper et al., 2007). In the structure model of PSGT with acarbose molecule, aromatic residues Y181, F185, and W219 were located in the putative acceptor binding subsites +2 and + 3 and interacted with the acarbose. The amino sugar of the acarviosin moiety in subsite +1 was located between Y270 (7.4 Å) and W219 (5.9 Å) while the glucose moiety in subsite +2 was located between Y181 (3.6 Å) and W219 (4.8 Å; Figure 6A). Therefore, the W219 residue would be essential for substrate binding at subsites +1 and + 2. The glucose moiety at the reducing end of the acarbose showed interaction with F185 (3.7 Å) at subsite +3.

To explore the functional roles of these amino acid residues in substrate binding and catalysis, we used site-directed mutagenesis to replace them with alanine. The resulting variants were purified and tested for their degradation activity against amylose in the presence of acceptors, such as glucose, maltose, and maltotriose (Figure 6B). The amylose degradation activity levels of rPSGT in the presence of glucose, maltose, and maltotriose acceptors were drastically reduced by replacing W219 with alanine. The degradation activity levels of Y181A in the presence of glucose, maltose, and maltotriose were reduced by 48, 67, and 64%, respectively. Furthermore, F185A had severely reduced amylose degradation activity in the presence of maltotriose whereas its degradation activity levels in the presence of maltose and glucose were similar to those of WT (Figure 6B). Therefore, the Y181 and F185 residues in the acceptor binding site of PSGT are vital to maltose and maltotriose binding, respectively.

### Comparing acceptor docking conformations of wild type and mutants

**Figure 7:**
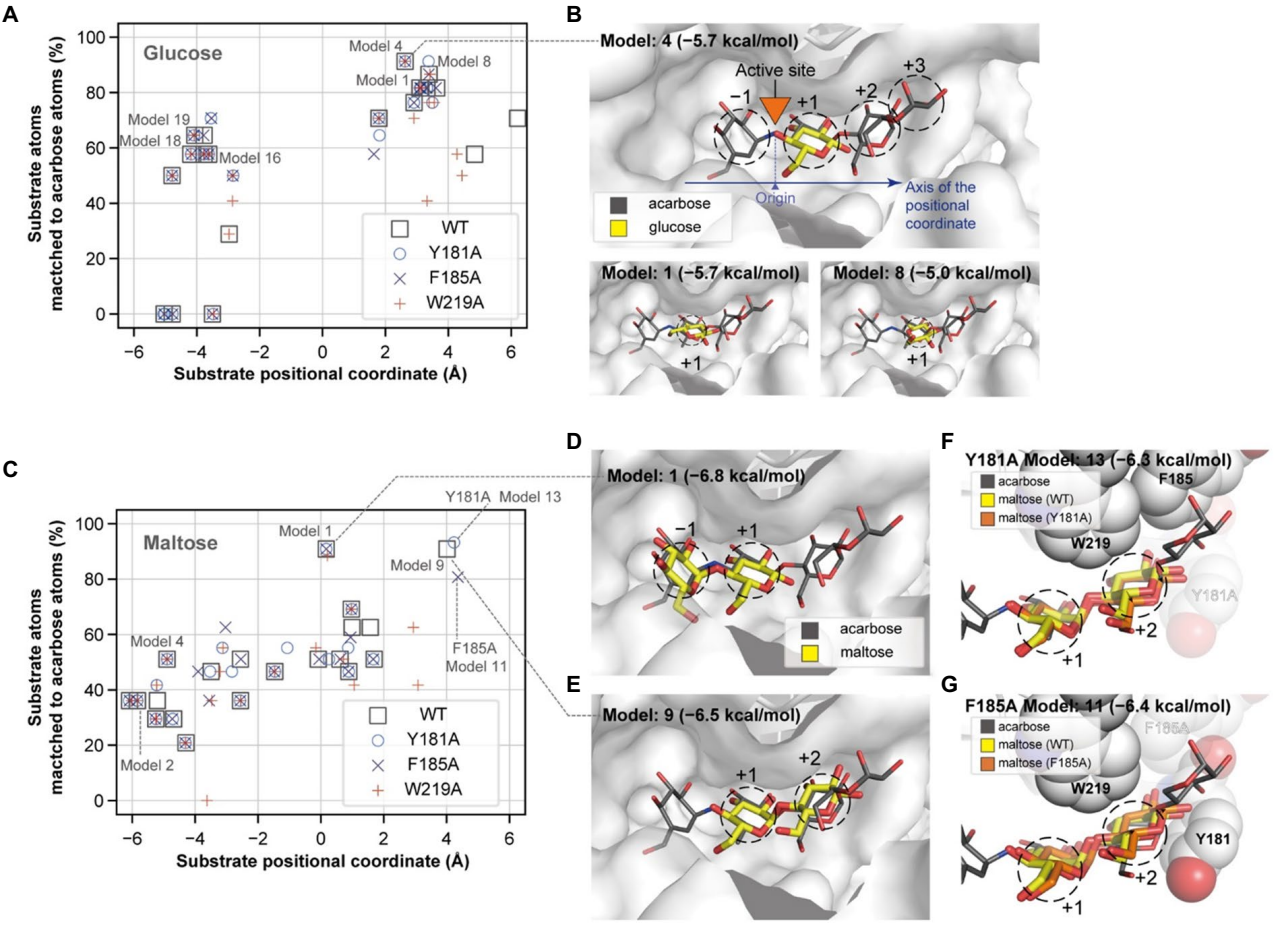
Glucose and maltose docking conformations. The docking positions of a glucose **(A)** and a maltose **(C)** molecule and their degrees of match to the inhibitor molecule are plotted for the wildtype and mutant PSGT structure models. The substrate positional coordinate in **(A,C)** is the projection of the center of heavy atom coordinates onto the axis of the substrate binding site, which is shown by the blue arrow in **(B)**. The position of the acarbose atom linking the two moieties in the −1 and + 1 positions in **(B)** was used as the origin of the coordinate. **(B,D,E)** The three-dimensional structures of the docking conformations are illustrated as yellow sticks on the surface of the enzyme, along with the structure of acarbose (in gray narrow sticks), AutoDock Vina docking scores, and the model number, which represents the rank of the docking score. **(F,G)** Docking conformation on the +1 and + 2 subsites of the Y181A and F185A variant structure models. Y181, F185, and W219 are shown as spheres, but the alanine substitute residues are displayed in translucent color.

To explain the role of the aromatic residues in acceptor selectivity, molecular docking simulations were implemented. Although none of the docking conformations for maltotriose and maltotetraose were similar to that of acarbose, we collected the appropriate docking conformation data for glucose and maltose. The docking conformations of the acceptors were clustered near 3 and − 4 Å of the substrate positional coordinate, which correspond to the +1 and − 1 subsites, respectively (Figures 7A,B). The conformation of the lowest energy docking model (Model 1) for glucose was placed at the +1 subsite, and the conformation of the fourth lowest energy docking model (Model 4) was similar to that of a glucose moiety of acarbose (Figure 7B). These results suggest that the glucose moiety would strongly bind to the −1 and + 1 subsites.

When maltose was docked on PSGT, the most energetically stable conformation was located on the −1 and + 1 subsites (Figures 7C,D). Maltose also docked at the +1 and + 2 subsites, although the docking conformation in the Y181A and F185A structure models slightly varied from that in the wild-type model (Figures 7E–G). The glucose moiety at the +2 subsite was moved toward the mutated Y181A residue in the variant structure, compared to that of the wild-type structure (Figure 7F). This suggests that the mutation allows the maltose to adopt a different conformation at the +2 subsite, and that the Y181 residue would increase the enzymatic activity by limiting the conformations of maltose to those allowing the transglycosylation reaction.

However, the conformation with binding at the +1 and + 2 subsites was not found in the docking simulation with the W219A structural model (Figure 7C). According to this structural model, W219 interacts with the acarbose glucose moieties in the +1 and + 2 subsite (Figures 6A, 7B); thus, the W219A mutation would abolish this interaction, leading to the loss of PSGT enzymatic activity.

## Discussion

The genera *Pyrococcus* and *Thermococcus* used GH13 and GH57 amylolytic enzyme, which enable these microorganisms to feed on starch and maltose. According to Lee et al., in the presence of starch and maltose in the growth medium, 4-α-glucanotransferase is significantly upregulated in *P. furiosus*, which harbors both GH13 and GH57 (Lee et al., 2006). However, some species such as *Pyrococcus* sp. NA2 and ST04 have only a gene belonging to GH57, and all the *Pyrococcus* species we studied possessed GH57 family genes (Supplementary Table S4). Thus, GH57 is likely to play an important role in carbohydrate utilization by the genus.

In the present study, we revealed the mode of action of GH57 4-α-GTase derived from *Pyrococcus* sp. ST04, which displayed significantly different enzymatic function from that of GH77 family 4-α-GTases identified from *Thermus brockianus*, *Thermus aquaticus*, *Thermus therm ophilus*, and *Pyrobaculum aerophilum*. GH77 family 4-α-GTase displays the highest activity toward maltotriose as the substrate and release glucose as the main product (Terada et al., 1999; Jung et al., 2011). In contrast, GH57 4-α-GTase, PSGT, showed low hydrolytic efficiency and low glucose yield when maltotriose was used as the substrate (Figure 2). To understand the different mode of action of PSGT, the product profile of PSGT disproportionation reaction was analyzed by HPAEC. We found that maltooligosaccharide_(n-1)_ and maltooligosaccharide_(n-2)_ were mainly produced from maltooligosaccharide_n_ (where *n* is the number of sugars) during the PSGT reaction (Figure 3). This hydrolysis pattern resembles that of the amylolytic enzyme from *Thermotoga maritima* (TMA; Lee et al., 2002) and maltodextrin glucosidase from *Escherichia coli* (MalZ; Dippel and Boos, 2005), in that they produce glucose and maltooligosaccharide_n-1_ from a maltooligosaccharide_n_ substrate. However, PSGT was also able to extend maltooligosaccharides *via* transglycosylation activity.

The unique product profile of the GH57 4-α-GTase PSGT can be attributed to the binding of substrates to the enzyme with different affinities. Pyzylase et al. and Kaper et al. reported that GH77 4-α-GTases from *Thermus* sp. have seven substrate binding subsites (+3 to −4; Przylas et al., 2000; Kaper et al., 2007). Similarly, the substrate binding site of GH57 4-α-GTase consists of at least nine subsites (+3 to −6; Imamura et al., 2003). However, glucose was produced as the main product by GH77 4-α-GTase but not by PSGT (Figure 5). The structure of GH77 4-α-GTase showed potential steric hindrance at the +2 subsite, which may lead to the production of a high concentration of glucose and generation of maltose only by transglycosylation (Jung et al., 2011). In contrast, PSGT, belonging to the GH57 family, lacks the steric hindrance at the +2 subsite, as a result of which the glucose moieties of maltooligosaccharides can bind to the three acceptor subsites (+1, +2, and + 3).

Moreover, our analyses demonstrated that the binding pattern of glucose moieties at these acceptor subsites is important to define the product profile. In the PSGT reaction with maltopentaose, glucose and maltose remained on the intermediate subsite and formed a glycosyl-enzyme complex, whereas maltotetraose and maltotriose were released from the active site pocket. To release maltotriose and maltotetraose in the PSGT reaction, maltopentaose must be localized to the subsites in the ranges of +4 to −1 and + 3 to −2 (Figure 5). The alternative substrate binding subsites resulted in different hydrolysis patterns, which have been previously observed in barley α-amylase (Gottschalk et al., 2001). Moreover, its maltodextrin utilization pattern was affected by its substrate binding preference. Maltose-producing enzymes such as maltogenic amylase also contain seven substrate binding subsites in their active site pockets. Their substrates exhibited high affinity for the +2 subsite, and production of maltose was favored over that of other maltooligosaccharides (Park et al., 2005; Kim et al., 2007).

The PSGT hydrolysis pattern suggested that maltotriose preferentially binds at an acceptor subsite. To demonstrate the binding affinity of maltotriose, *p*-nitrophenyl-α-hexopyranoside (*p*NPG6) was used as the sole substrate for the enzyme reaction. PSGT produced *p*NPG2, which is equivalent to maltotriose, on hydrolysis, and generated *p*NPG10 as the transglycosylated product. This indicates that the hydrolytic cleavage occurred when the three acceptor subsites were occupied. Furthermore, it is likely that the maltotetraose produced by the hydrolysis of *p*NPG6 would be retained in PSGT and transglycosylated to *p*NPG6 to produce pNPG10 (Supplementary Figure S2). This preference was also reflected in the kinetic analysis of substrate affinity for the acceptor binding site (especially +3), with the *K*_M_ value gradually reducing in the order glucose > maltose > maltotriose (Table 2). It also suggested that when the substrate molecules bind on the PSGT, the molecules preferentially cover the acceptor binding site from +1 to +3 (Figure 5).

This acceptor preference has also been observed in GH57 4-α-GTase from *T. onnurineus*, which exhibited that maltose is strong acceptor molecules compared with glucose (Kaila and Guptasarma, 2019). It concluded that the binding affinity of GH57 4-α-GTase, PSGT, was clearly different from that of GH77 4-α-GTase, which captured acarbose at the subsites −1 to −3 (Figure 5; Przylas et al., 2000).

**Figure 8:**
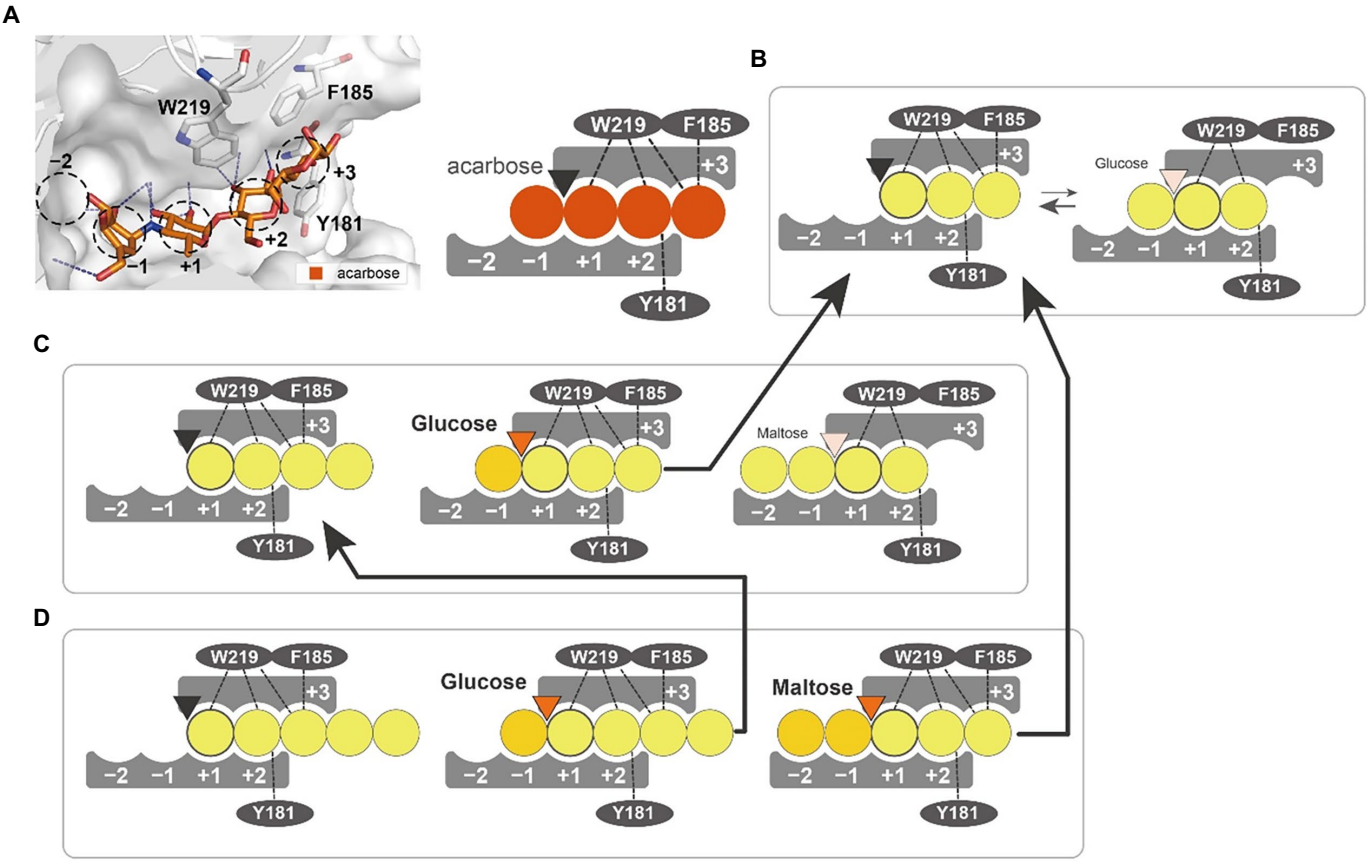
Substrate interaction of PSGT. **(A)** Glucose moiety-binding sites of PSGT, with acarbose. The −1, +1, +2, and + 3 positions were determined from the structure of acarbose in the *Thermococcus litorallis* 4-α-glucanotransferase (PDB ID = 1K1Y). The glucose binding sites of the enzyme are illustrated as a figurative diagram of the enzyme and substrate. The interactions of ligands with Y181, F185 and W219 are illustrated as dashed lines. **(B–D)** Interaction of maltotriose **(B)**, maltotetraose **(C)**, and maltopentaose **(D)** with PSGT. The PSGT active site is depicted as a triangle and colored by its enzymatic status: black (inactive), orange (active), and pale orange (weakly active).

We suggest a putative action model based on molecular dynamics simulation and mutant analysis to explain the generation of different products from substrates of varying length (Figure 8). In the PSGT structure model, three aromatic amino acid residues (Y181, F185, and W219) surround the putative acceptor binding site. Interestingly, the W219 is an amino acid belonging to CSR5 of the GH57 family (Supplementary Figure S3). Tryptophan residue is highly conserved in the sequences of 4-α-GTase and branching enzyme (Martinovicova and Janecek, 2018). Although the other two amino acids (Y181 and F185) were not located in the GH57 family specific five conserved regions, the multiple sequence alignment of GH57 4-α-GTases showed that these amino acids were commonly found in the specificity of 4-α-GTase (Supplementary Figure S4). Interestingly, structural comparison with GH57 maltose-forming amylase and Branching enzyme suggested that the location of Y181 and F185 residues were similarly overlapped with F218 of GH57 maltose-forming amylase and F270 of Branching enzyme (Supplementary Figure S5). F218 residue of maltose-forming amylase was known as a modulator of substrate binding, whereas F270 residue of Branching enzyme was gate-keepers on the entrance of substrate binding (Santos et al., 2011; Park et al., 2014).

The enzymatic properties of Y181A and F185A mutants demonstrated that these two amino acids stabilize the binding of acceptor molecules, but have different roles in substrate interaction. The Y181 residue interacted with the acceptor molecules at the +2 subsite, which limited their binding conformations to those that enable the transglycosylation reaction. Therefore, the Y181A mutation reduced the amylose degradation activity of the enzyme in the presence of maltose (Figure 6B). However, the enzymatic activity was reduced when maltotriose was used as the acceptor, which suggests that the conformation restriction at the +2 subsite would be required for the stabilizing interaction at the +3 subsite. In contrast, the F185A mutation reduced the amylose degradation activity specifically with maltotriose (Figure 6B), demonstrating that the Phe residue stabilizes the glucose moiety binding at the +3 subsite. According to the glucose docking simulation, the glucose was favorably docked on the +1 subsite. Together, these results indicate that the first glucose moiety of an acceptor molecule would most strongly bind to the +1 subsite; the adjacent moiety would then bind to the +2 subsite, which would allow the next moiety to stably bind to the +3 subsite.

The W219A mutation abolished the enzymatic activity, suggesting its critical involvement in the enzymatic reaction. Our docking simulation revealed that this mutation would weaken the substrate binding at the +1 and +2 subsites (Figures 7C,E). Tang et al. reported that the W221 residue was implicated in the transglycosylation activity of *P. furiosus* 4-α-GTase (Tang et al., 2006). Therefore, the W219 residue in PSGT may also be directly associated with its catalytic activity.

Our proposed model explained that rPSGT utilized maltooligosaccharides in a manner distinct from that of GH77 4-α-GTases (Figures 5, 8). It resulted in different disproportionation ability by acceptor types. Table 3 compares the properties of the GH77 and GH57 4-α-GTases. These unique properties of GH57 4-α-GTases compared to those of GH77 were probably affected by relative differences in their cytoplasmic function. *Pyrococcus* sp. ST04 has no GH13-type amylolytic enzymes such as cyclodextrinase, which was found only in *P. furiosus* and *P. yayanoshii*. Further studies on the PSGT reaction mechanism and its associated metabolic pathway may reveal details of the glucan utilization system of hyperthermophile *Pyrococcus*, which can be exploited to improve the glycosylation process.

**Table 3:** Comparison of biological function between two glucanotransferase GH family.

Property	GH family 77	GH family 57
Core structure	(β/α)_8_	(β/α)_7_
Reaction mechanism	Retaining	Retaining
Best substrate	Maltotriose	N.A^‡^ ^†^
Major product from G5	Glucose	Maltotetraose
Best acceptor	Glucose	Maltotriose
Transglycosylation factor^*^		
Glucose	54 ± 6	4.6 ± 0.2
Maltose	28 ± 2	12.2 ± 1.4
Maltotriose	N.A^‡^	18.3 ± 1.5

‡ N.A, Not available.

† The substrate specificity of GH family 57 amylomaltase was unable to be calculated.

* Transglycosylation factor was defined as the ratio of amylose degradation with acceptor relative to that without acceptor. Transglycosylation factor of GH family 77 4-α-GTase was determined for only TBGT with 10 mM acceptors (Jung et al., 2011), whereas transglycosylation factor of PSGT was calculated in the presence of 5 mM acceptors.

## Data availability statement

The original contributions presented in the study are included in the article/Supplementary material; further inquiries can be directed to the corresponding author.

## Author contributions

J-HJ and C-SP performed research and wrote the manuscript. SH, M-KK, and E-JW contributed to the structural analysis. EJ, D-HS, and JH participated in data analysis. The manuscript was written through the contribution of all authors. All authors contributed to the article and approved the submitted version.

## Funding

This research was supported by the KAERI institutional R&D Program (Project No. 523430-22) funded by Ministry of Science and ICT (MIST), South Korea.

## Conflict of interest

The authors declare that the research was conducted in the absence of any commercial or financial relationships that could be construed as a potential conflict of interest.

## Publisher’s note

All claims expressed in this article are solely those of the authors and do not necessarily represent those of their affiliated organizations, or those of the publisher, the editors and the reviewers. Any product that may be evaluated in this article, or claim that may be made by its manufacturer, is not guaranteed or endorsed by the publisher.

## Footnotes

^1^ https://www.ncbi.nlm.nih.gov/

^2^ www.cazy.org
