# Supplementary material for: Acceptor dependent catalytic properties of GH57 4-α-glucanotransferase from Pyrococcus sp. ST04
Source: Front Microbiol. 2022 Oct 6;13:1016675. doi: 10.3389/fmicb.2022.1016675 (PMC9582752; doi:10.3389/fmicb.2022.1016675)
Supplement: Supplementary file 1 [file Data_Sheet_1.docx]

**Frontiers in Microbiology**

**Supplementary Material**

**for**

Acceptor dependent catalytic properties of GH57 4-α-glucanotransferase from *Pyrococcus* sp. ST04

Jong-Hyun Jung^1^, Seungpyo Hong^2^, Eun Jung Jeon^3^, Min-Kyu Kim^1^, Dong-Ho Seo^4^, Eui-Jeon Woo^5^, James F. Holden^6^ and Cheon-Seok Park^7^*

^1^Radiation Research Division, Korea Atomic Energy Research Institute, Jeongeup, South Korea

^2^Department of Molecular Biology, Jeonbuk National University, Jeonju, South Korea

^3^Department of Chemical and Biomolecular Engineering, Korea Advanced Institute of Science and Technology (KAIST), Daejeon, South Korea

^4^Department of Food Science and Technology, Jeonbuk National University, Jeonju, South Korea

^5^Korea Research Institute of Bioscience and Biotechnology (KRIBB), Daejeon, South Korea

^6^Department of Microbiology, University of Messachusetts, Amherst, MA, USA

^7^Department of Food Science and Biotechnology and Institute of Life Science and Resources, Kyung Hee University, Yongin, South Korea

*** Correspondence:**

^†^Corresponding author:

Mailing address: *Department of Food Science and Biotechnology and Institute of Life Science and Resources*, Kyung Hee University, Yongin, Korea

Phone: 82-31-201-2631; Fax: 82-31-204-8116; E-mail: cspark@khu.ac.kr

**Growth medium conditions**

*Pyrococcus* sp. strain ST04 was grown anaerobically at 92°C in serum bottles sealed with a butyl rubber stopper and flushed with N_2_ : CO_2_ (80:20) gas. The medium was derived from DSM medium 141 (www.dsmz.de) and was composed of the following in distilled water (per liter): 18 g NaCl, 4 g MgCl_2_•6H_2_O, 3.45 g MgSO_4_·7H_2_O, 0.335 g KCl, 0.25 g NH_4_Cl, 0.14 g CaCl_2_·2H_2_O, 0.14 g K_2_HPO_4_, 0.5 g yeast extract (Difco, vitamin B_12_ fortified), 1 g NaHCO_3_, 10 ml of DSM medium 141 trace element solution (Table M1), 10 ml of DSM medium 141 vitamin solution (Table M2), 1 ml of 0.2% (NH_4_)_2_Fe(SO_4_)_2_ – 0.2% (NH_4_)_2_Ni(SO_4_)_2_ solution, 0.10 ml of 100 mM Na_2_WO_4_·2H_2_O – 100 mM Na_2_SeO_4_ solution, and 0.25 mg resazurin. To reduce the media, 0.025% (wt vol^-1^) each of cysteine-HCl·H_2_O and of Na_2_S·9H_2_O were added. The pH of the medium was adjusted to 6.80 ± 0.05 (room temperature) unless otherwise stated. After reduction and pH adjustment, 1 ml of 1 M potassium phosphate solution (pH 6.8) was added as a pH buffer. For growth on sulfur, 0.1% (wt vol^-1^) of elemental sulfur (Sigma Chemical Co., St Louis, MO, U.S.A.) was added to the media.

**Supple Table S1**. Composition of DSM medium 141 trace element solution (per liter of dH_2_O)

| Nitrilotriacetic acid | 1.500 g |
| --- | --- |
| MgSO_4_ · 7H_2_O | 3.000 g |
| MnSO_4_ · 2H_2_O | 0.500 g |
| NaCl | 1.000 g |
| FeSO_4_ · 7H_2_O | 0.100 g |
| CoSO_4_ · 7H_2_O | 0.180 g |
| CaCl_2_ · 2H_2_O | 0.100 g |
| ZnSO_4_ · 7H_2_O | 0.180 g |
| CuSO_4_ · 5H_2_O | 0.010 g |
| KAl(SO_4_)_2_ · 12H_2_O | 0.020 g |
| H_3_BO_3_ | 0.010 g |
| Na_2_MoO_4_ · 2H_2_O | 0.010 g |
| NiCl_2_ · 6H_2_O | 0.025 g |

Firstly, NTA is dissolved in solution adjusted to pH 6.5, and then add other minerals.

pH of trace element solution is pH 7.0

**Supple Table S2**. Composition of DSM medium 141 vitamin solution (per liter of dH_2_O)

| Biotin | 2.0 mg |
| --- | --- |
| Folic acid | 2.0 mg |
| Pyridoxine-HCl | 10.0 mg |
| Thiamine-HCl · 2H_2_O | 5.0 mg |
| Riboflavin | 5.0 mg |
| Nicotinic acid | 5.0 mg |
| D-pantothenate | 5.0 mg |
| Vitamin B_12_ | 0.1 mg |
| p-Aminobenzoic acid | 5.0 mg |
| Lipoic acid (6,8-thioctic acid) | 5.0 mg |

**Supple Table S3**. **The sequences of primers for site-directed mutagenesis**

| **Mutants** | **Name** | **Sequences** |
| --- | --- | --- |
| Y181A | Y181A_F | 5'-cgttgtttttccaatcgatgaaaagctcagagctctaatcccctttagg-3' |
|  | Y181A_R | 5'-cctaaaggggattagagctctgagcttttcatcgattggaaaaacaacg-3' |
| F185A | F185A_F | 5'-gctcagatatctaatccccgctaggcccgttgaacaggtt-3' |
|  | F185A_R | 5'-aacctgttcaacgggcctagcggggattagatatctgagc-3' |
| W219A | W219A_F | 5'-catgacgatggagaaaaatttggaatagcgcccggtacttacg-3' |
|  | W219A_R | 5'-cgtaagtaccgggcgctattccaaatttttctccatcgtcatg-3' |

**Supple Table S4**. Glycoside hydrolase family enzymes in the *Pyrococcus* sp. ST04

| Strains | Glycoside hydrolase family  (α-linkage active)* | | | | | |
| --- | --- | --- | --- | --- | --- | --- |
|  | 13 | 38 | 57 | 65 | 122 | 130 |
| *Pyrococcus yayanosii* CH1 | 3 | - | 3 | - | 1 | - |
| *Pyrococcus furiosus* DSM 3638 | 3 | - | 4 | - | 1 | 1 |
| *Pyrococcus horikoshii* OT3 | - | 1 | 3 | 1 | 1 | 1 |
| *Pyrococcus abyssi* GE5 | - | - | 3 | - | 1 | 1 |
| *Pyrococcus* sp. NA2 | - | - | 1 | - | 1 | - |
| ***Pyrococcus* sp. ST04** | - | 1 | 3 | 1 | 1 | 1 |

*GH family 13 consist of α-amylase superfamily; GH family 38 contains α-mannosidase; GH family 57 consist of amylomaltase, branching enzyme, amylopullulanase, α-galactosidase, and α-amylase. GH family 65 contains disaccharide phosphorylases; GH family 122 contains α-glucosidase; and GH family 130 contains phosphate α-D-mannosyltransferase


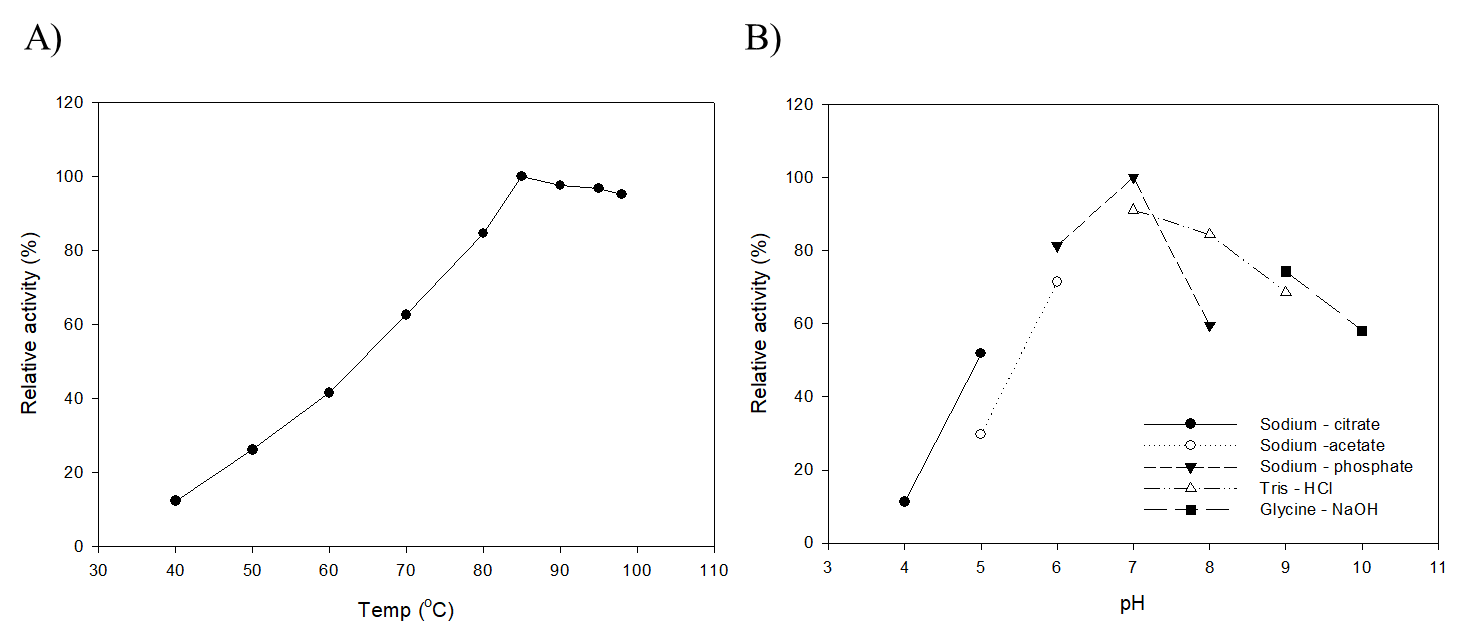


**Supple Figure S1**. **Optimum temperature and pH of recombinant PSGT on the amylose degradation activity**. (A) For determination of optimum temperature, the reactions were performed with 0.5% (w/v) amylose and 0.097 mg/mL purified rPSGT at various temperature ranging 40~99 ^o^C. (B) The optimum pH was determined using 50 mM Sodium acetate (pH 4-5), 50 mM sodium citrate (pH 5-6), 50 mM sodium phosphate (pH 6-8), 50 mM Tris-HCl (pH 7-9), and 50 mM glycine-NaOH (pH 9-10). The reaction mixture containing 0.5% amylose and 0.097 mg/mL was incubated at 85^o^C in various pH conditions. The amylose degradation was measured using iodine method at 660 nm absorbance


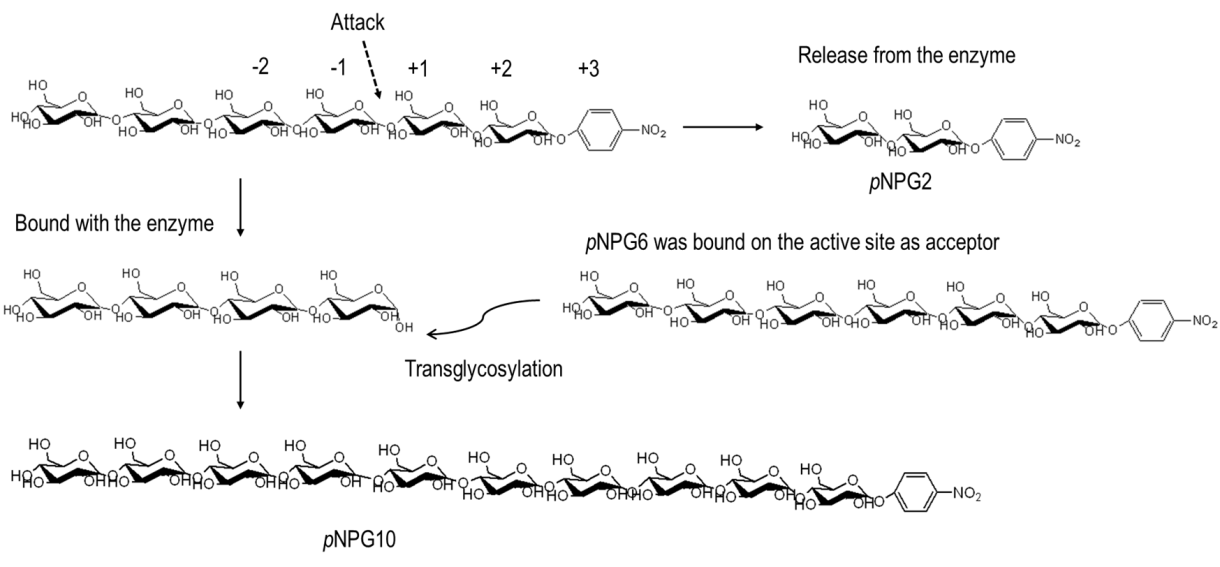


**Supple Figure S2**. Scheme of action mode of recombinant PSGT on pNPG10


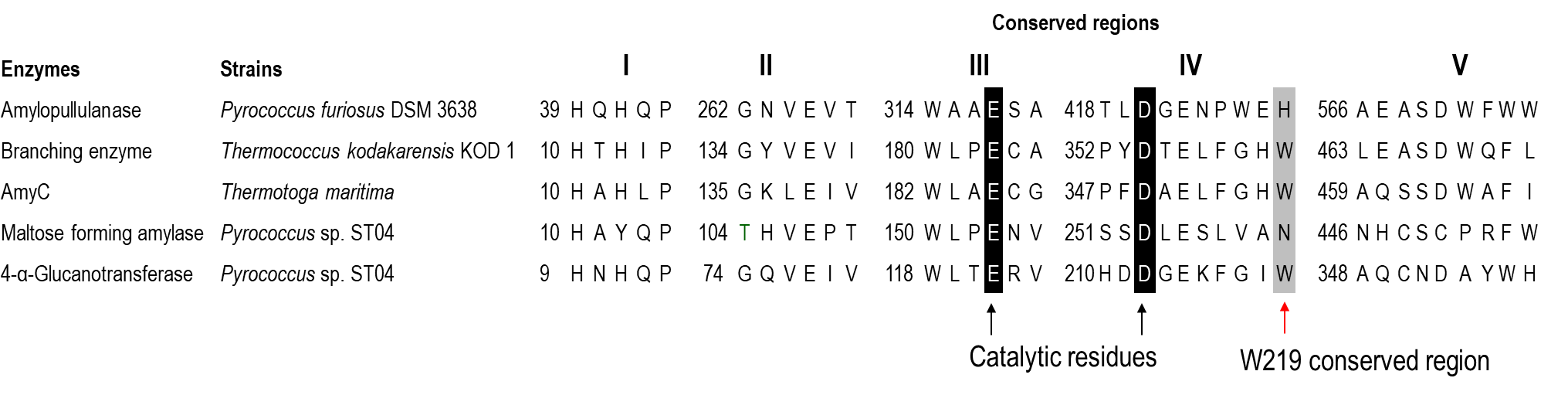


**Supple Figure S3**. **Multiple alignment of conserved region (CSR) sequence in Glycoside hydrolase 57 family enzyme.** Black arrow indicated that catalytic residue location. Red arrow mean location of W219 site of PSGT


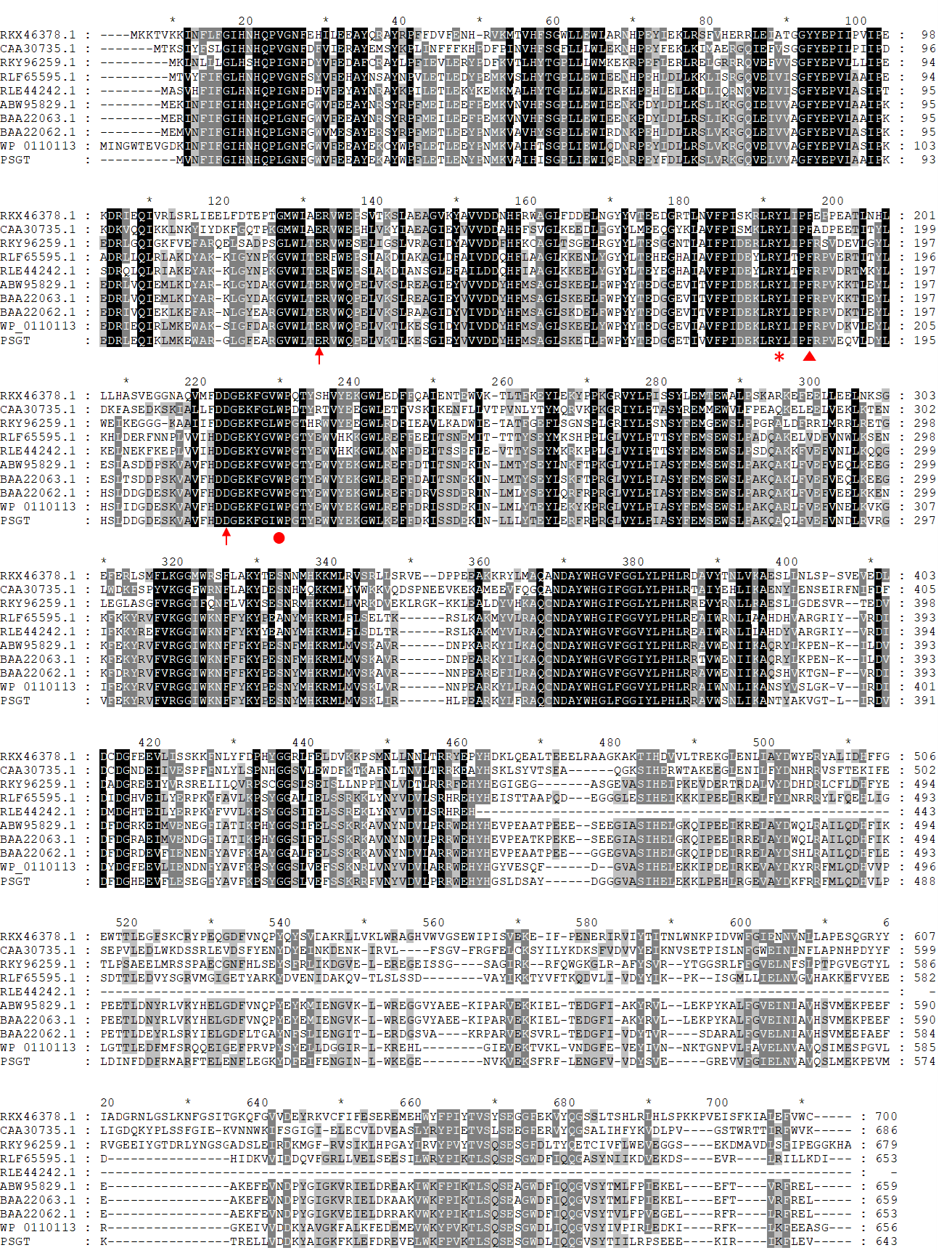


**Supple Figure S4**. **Multiple sequence alignment of 4-α-GTase sequences**. RKX46378.1: 4-α-glucanotransferase from *Thermotogae* bacterium, CAA30735.1: 4-α-glucanotransferase from *Dictyoglomus thermophilum*, RKY96259.1: 4-α-glucanotransferase from *Candidatus Hydrothermae* bacterium, RLF65595.1 4-α-glucanotransferase from *Thermoplasmata* archaeon, RLE44242.1: 4-α-glucanotransferase, partial from *Candidatus Woesearchaeota* archaeon, ABW95829.1: cyclodextrin glucanotransferase from *Archaeoglobus fulgidus*, BAA22063.1: 4-α-glucanotransferase from *Thermococcus litoralis*, BAA22062.1: 4-α-glucanotransferase from *Thermococcus kodakaraensis* KOD1, WP_011011387.1 4-α-glucanotransferase from Pyrococcus furiosus, PSGT : 4-α-glucanotransferase from *Pyrococcus* sp. ST04. Red arrow indicated catalytic residues (Glu, and Asp, *: Y181 position of PSGT, ▲: F185 position of PSGT and ●: W219 position of PSGT


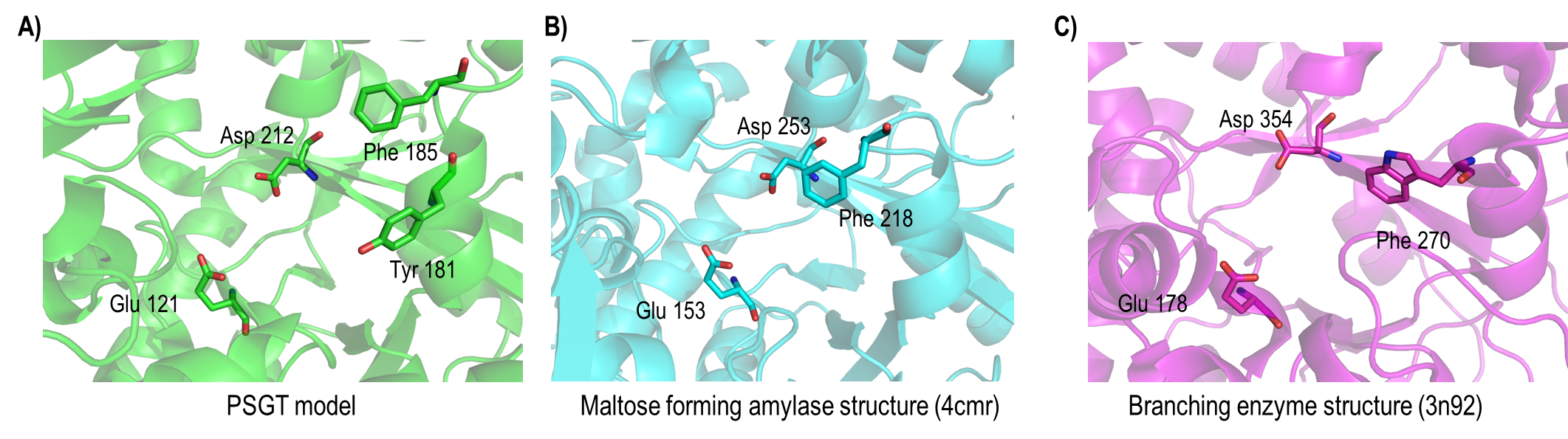


**Supple Figure S5**. **Structural comparison of conserved catalytic and acceptor binding subsite amino acids in GH57 family 4-α-GTase (A), maltose-forming amylase (B) and Branching enzyme (C).** Structure view of 4-α-GTase was constructed based on PSGT model, whereas visualization of maltose forming amylase and branching enzyme were displayed using the 3D structure of maltose forming amylase from *Pyrococcu*s sp. ST04 (4cmr) and Branching enzyme from *Thermococcus kodakaraensis* KOD1 (3n92)
